# Impaired connectivity within neuromodulatory networks in multiple sclerosis and clinical implications

**DOI:** 10.1007/s00415-020-09806-3

**Published:** 2020-03-26

**Authors:** Antonio Carotenuto, Heather Wilson, Beniamino Giordano, Silvia P. Caminiti, Zachary Chappell, Steven C. R. Williams, Alexander Hammers, Eli Silber, Peter Brex, Marios Politis

**Affiliations:** 1 Neurodegeneration Imaging Group, Institute of Psychiatry, Psychology and Neuroscience, King’s College London, London, UK; 2 Multiple Sclerosis Clinical Care and Research Centre, Department of Neuroscience, Federico II University, Naples, Italy; 3 Neurodegeneration Imaging Group, University of Exeter Medical School, London, UK; 4 Institute of Psychiatry, Psychology and Neuroscience, Institute of Psychiatry, King’s College London, London, UK; 5 King’s College London and Guy’s and St Thomas’ PET Centre, School of Biomedical Engineering and Imaging Sciences, Faculty of Life Sciences and Medicine, King’s College London, St Thomas’ Hospital, London, UK; 6 Department of Neurology, King’s College Hospital NHS Foundation Trust, London, UK

**Keywords:** Functional connectivity, Graph theory, Multiple sclerosis, Neurotransmitter, Resting-state fMRI

## Abstract

There is mounting evidence regarding the role of impairment in neuromodulatory networks for neurodegenerative diseases, such as Parkinson’s and Alzheimer’s disease. However, the role of neuromodulatory networks in multiple sclerosis (MS) has not been assessed. We applied resting-state functional connectivity and graph theory to investigate the changes in the functional connectivity within neuromodulatory networks including the serotonergic, noradrenergic, cholinergic, and dopaminergic systems in MS. Twenty-nine MS patients and twenty-four age- and gender-matched healthy controls performed clinical and cognitive assessments including the expanded disability status score, symbol digit modalities test, and Hamilton Depression rating scale. We demonstrated a diffuse reorganization of network topography (*P* < 0.01) in serotonergic, cholinergic, noradrenergic, and dopaminergic networks in patients with MS. Serotonergic, noradrenergic, and cholinergic network functional connectivity derangement was associated with disease duration, EDSS, and depressive symptoms (*P* < 0.01). Derangements in serotonergic, noradrenergic, cholinergic, and dopaminergic network impairment were associated with cognitive abilities (*P* < 0.01). Our results indicate that functional connectivity changes within neuromodulatory networks might be a useful tool in predicting disability burden over time, and could serve as a surrogate endpoint to assess efficacy for symptomatic treatments.

**Electronic supplementary material:**

The online version of this article (10.1007/s00415-020-09806-3) contains supplementary material, which is available to authorized users.

## Introduction

Focal demyelination in multiple sclerosis (MS) leads to the disruption of the axon–myelin unit and sub-sequential neuronal damage [1]. Loss of pre-synaptic neurons causes reduced neurotransmitter release at the synaptic cleft resulting in disrupted functional connectivity among distant brain regions [2]. Post-mortem studies in MS reported a reduced synaptic density and impaired axonal transport, synaptic plasticity, glutamate neurotransmission, and glutamate homoeostasis [3]. Demyelination, axonal loss, and synaptic pathology cause a dysfunctional communication among brain regions. Neurons throughout the brain communicate through the release of neurotransmitters, subsequently modulating neuronal activity. Several studies have outlined neurotransmitter dysfunction in neurodegenerative disorders, such as Parkinson’s, Alzheimer’s, and Huntington’s disease, involving mainly the dopaminergic, serotonergic, and cholinergic networks. Network impairment in such neurodegenerative disorders has been selectively associated with neurological symptoms such as cognitive, motor, and non-motor symptoms [4–8]. Tools to measure impaired neurotransmitter networks integrity require either the application of PET or complex MRI methodology with contrast injection. For this reason, since MS is not primarily a neurotransmitter disorder, the precise involvement of neuromodulatory networks in MS has not been fully elucidated and existing evidence is scarce. Previous studies reported that a reduced glutamate and gamma-aminobutyric acid concentration throughout several brain areas in MS patients measured through magnetic resonance spectroscopy in the brain correlated with clinical disability [9–11].

Resting-state functional magnetic resonance imaging (fMRI) is able to explore connectivity in predefined networks as for example the default mode network, the visual network, and the motor network. In MS, patients could experience either a decrease or an increase in functional connectivity [12–16]. In addition, resting-state fMRI might also depict network topography changes, through graph theoretical analyses. Graph theory is a mathematical representation of the brain connections, consisting of a set of nodes (corresponding to cerebral regions) and edges (corresponding to functional or structural connection) interposed between them. The topography of the network can be described using different measures such as clustering coefficient, path length, and efficiency measures to characterize the ‘small-world’ properties of brain networks or degree and betweenness centrality to identify the crucial areas within the network [17].

Recent evidence suggests that motor and cognitive decline in neurodegenerative disorders, such as Parkinson’s and Alzheimer’s disease, selectively depends upon neurotransmission integrity of dopaminergic and cholinergic networks, respectively [18, 19]. We hypothesized that demyelination and axonal loss randomly occurring throughout the brain, in MS patients, might cause dysfunctional activity and, hence, impaired functional connectivity (FC) in neuromodulatory networks. Furthermore, clinical outcomes in MS, such as depressive symptoms or cognitive impairment, could depend on the integrity of selected neuromodulatory networks as has been observed in other neurological disorders, such as the selective loss of dopaminergic neurons in Parkinson’s disease leading to motor, or the loss of cholinergic neurons in Alzheimer’s disease leading to cognitive dysfunction [18, 19]. Therefore, we aimed at investigating FC and network topography changes within the serotonergic, noradrenergic, cholinergic, and dopaminergic networks in MS, and to explore their relationship with both physical and cognitive disability.

## Materials and methods

### Subjects

Twenty-nine patients with a definite diagnosis of relapsing–remitting MS according to the 2010 revised McDonalds criteria [20] and twenty-four healthy controls (HCs) were recruited from the specialist clinics at King’s College Hospital NHS Foundation Trust and through advertisement. All participants had no history of other neurological or psychiatric disorders, and were successfully screened to undertake MRI scanning according to the standard MRI scanning safety criteria (https://www.mrisafety.com). For each subject, clinical disability was assessed using the Expanded Disability Status Scale (EDSS). Multiple Sclerosis Severity Score (MSSS) was calculated as measure of disease severity [21]. Cognitive function was assessed with the symbol digit modalities test (SDMT). This test was chosen among other cognitive tests, because it showed a high sensibility and specificity in detecting cognitive impairment in MS [22]. The Hamilton depression rating scale (HRSD) was used to assess neuropsychiatric symptoms. The study was approved by the London-South East Research Ethics Committee. Written informed consent was obtained from all study’s subjects prior to inclusion in the study in accordance to the Declaration of Helsinki. Data will be made available via a request to the authors after a formal data sharing agreement.

### MRI acquisition

MRI images were acquired using a 3-T Magnetom Biograph mMR PET/MR hybrid scanner (Siemens, Erlangen, Germany). MRI sequences included three-dimensional magnetization-prepared rapid gradient‐echo T1 (three-D-T1w MPRAGE, TR [repetition time]: 1700 ms, TE [echo time]: 2.63 ms, TI [inversion time]: 900 ms, flip angle: 9°, voxel size: 1.1 × 1.1 × 1.1 mm), turbo spin-echo T2-weighed images (T2w TSE, TR: 3200 ms, TE: 409 ms, TI: 900 ms, voxel size: 1 × 1 × 1mm); and fast fluid-attenuated inversion recovery (FLAIR, TR: 5000 ms, TE: 499 ms, TI: 1800 ms, voxel size: 1 × 1 × 1mm). In addition, resting-state fMRI scans were acquired using an echo-planar imaging sequence (TR: 3000 ms, TE: 30 ms, voxel size: 3 × 3 × 3mm, echo-planar imaging factor: 64, field of view: 192 mm, number of slices: 36; number of volumes: 240, flip angle: 90°).

### MR image processing

For each subject, T1 and T2 lesions were manually defined through Analyze medical imaging software (version 12, Mayo Foundation AnalyzeDirect).

T1 lesions mask was used to perform a ‘lesion filling’ procedure [23] on the 3D-T1w MPRAGE. We applied SIENAX [24] to estimate cortical gray matter volume, normalized for subject head size. Multi-atlas propagation with enhanced registration approach (MAPER) [25] method was applied to create the subject-specific brain segmentation. This robust, automated technique segments subjects’ structural T1w MRI into 95 anatomical regions with high accuracy and is applicable even to subjects with significant cortical atrophy and/or ventriculomegaly.

Structural and fMRI data were further processed analyzed through SPM12 software (Statistical Parametric Mapping version 12; https://www.fil.ion.ucl.ac.uk/spm/software/spm12/) and the CONN toolbox version 17a [26]. Briefly, T1 lesion-filled scans and gray matter regions of interest map were normalized into the Montreal Neurological Institute (MNI) 152 space atlas. FMRI data were realigned and co-registered to the structural images, normalized to the MNI-152 space atlas, and then smoothed, using 6 mm full width half max isotropic Gaussian kernels band-pass filtering was performed with a frequency window of 0.008–0.09 Hz. Signal contributions from micro-head movements were corrected using the image ‘scrubbing’ ART method implemented in CONN toolbox. Outlier time points were included as covariates in denoising, and first-level general linear model along with motion parameters.

### Networks’ definition

**Fig. 1:**
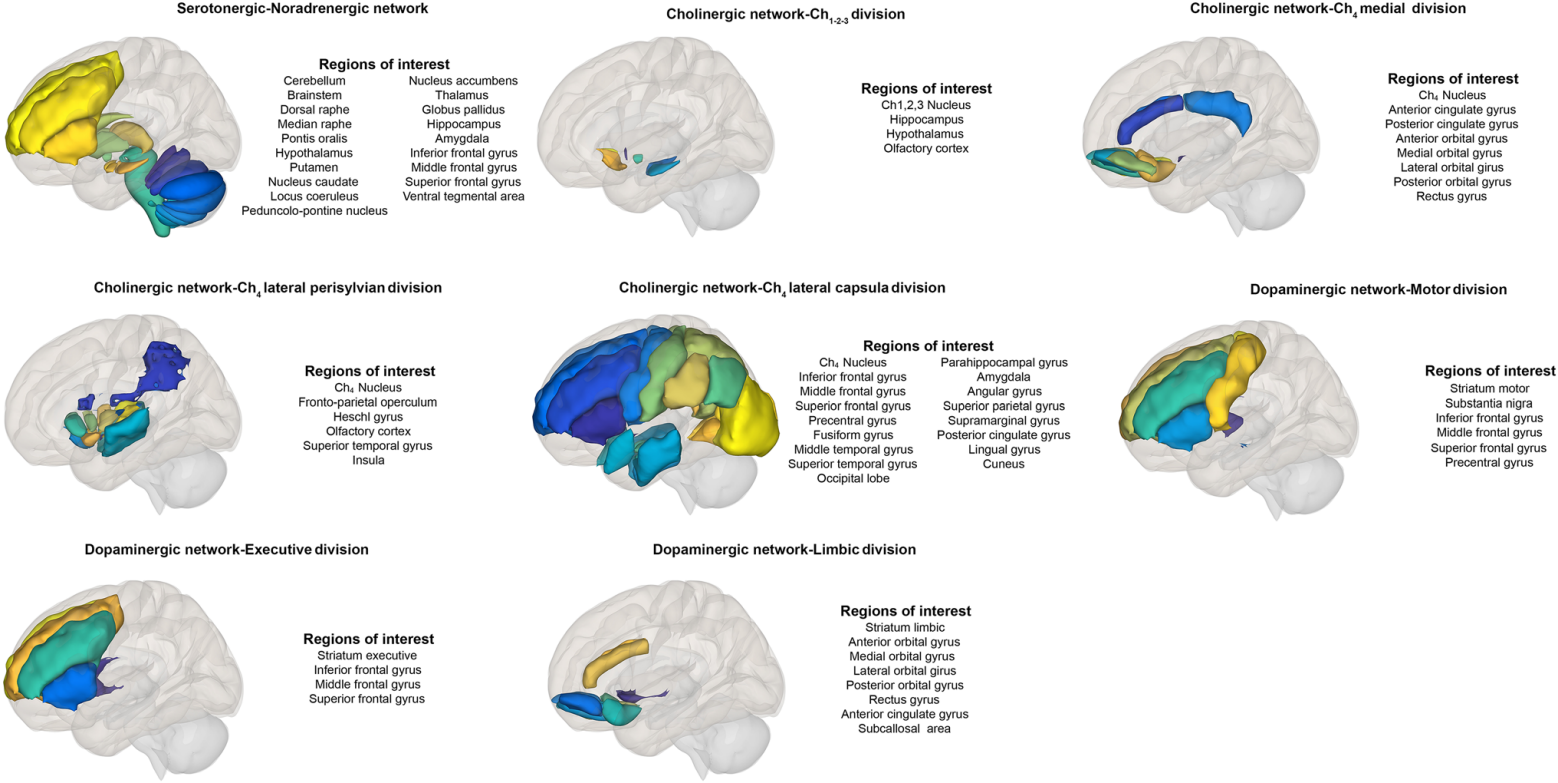
Neuromodulatory networks. Cortical and subcortical projections of the neuromodulatory networks assessed in this study with the description of the anatomical regions selected as regions of interest

We performed a region-of-interest-based seed-to-seed connectivity analysis within four selected neuromodulatory networks: the serotonergic, the noradrenergic, the cholinergic, and the dopaminergic network. Network maps were created including anatomic regions of the central nervous system with afferent and efferent neurons using selected neuromodulatory molecules. To maximize the precision of the anatomical localization, we selected brain regions from subject-specific brain segmentation with the MAPER approach [25]. This approach allows brain segmentation in native space. When unavailable on the subject-specific brain segmentation, brain regions were selected from standard atlases to complete the network. For the detailed description of networks’ anatomy, please see Fig. 1 and Supplementary Appendix A-1. Briefly, the serotonergic network was created following Strac and colleagues, and Jacobs and Azmitia anatomical description [27, 28]. The cholinergic network was created following Selden and colleagues anatomical description, including four divisions: the Ch_1-2–3_ division, the Ch_4_ medial division, the Ch_4_ lateral perisylvian division, and the Ch_4_ lateral capsula division [29]. For the noradrenergic network, taking into account Samuels and colleagues anatomical description and highly overlapping regions within the serotonergic network (noradrenergic network includes the locus coeruleus, pedunculopontine nucleus, and ventral tegmental area in addition to the serotonergic network), we decided to merge the two networks creating a serotonergic–noradrenergic network to avoid ROI overlapping [30]. The dopaminergic network was created following Tziortzi and colleagues anatomical description, including three divisions: the dopaminergic executive division, the dopaminergic limbic division, and the dopaminergic motor division excluding the ventral tegmental area already included in the serotonergic–noradrenergic network to avoid ROI overlapping [31]. For each region of interest, we verified that the volume for each region was greater than the full width half max of the scanner resolution to avoid blurring effect. For each subject, we created a region of interest-to-region of interest serotonergic, cholinergic, noradrenergic, and dopaminergic functional connectivity matrix in MNI space.

### Graph theory measures

For each network matrices, we performed a network topographic analysis through CONN functional connectivity toolbox [26]. We used a set of functional connectivity indices, namely the global efficiency, the local efficiency, the betweenness centrality, the average path length, the clustering coefficient, and the degree [17]. Global efficiency is a measure of network integration describing the information flow over the whole network. Local efficiency is the distance between the nodes and is a measure of network integration. Betweenness centrality is a measure of network centrality; it corresponds to the fraction of all the shortest paths in a network over a given node. Nodes with higher betweenness centrality participate in a large number of short paths. Average path length corresponds to the shortest distance between pairs of nodes, and is a measure of network efficiency of information transfer within the network. An increased average path length reflects the inability of the network to transfer information in parallel. Clustering coefficient is a measure of network organization reflecting the number of connections between the neighbor’s nodes. Degree is a measure of centrality and reflects the number of connections for each node to all other nodes in the network. A higher degree means a greater influence of the corresponding region in the network. To extract the best indices of network organization, network edges were adjusted with a threshold for a cost higher than 0.15 on a two-sided test for the adjacency matrix [32].

### Statistics

Statistical analyses were performed through CONN functional connectivity toolbox using a seed-to-seed approach in SPM. Between-group analysis was performed using two-tailed *t* tests to compare the resting-state functional connectivity and graph theory measures change between MS patients and HCs for each network. Linear regression analysis was also performed to investigate the association between clinical disability and both the resting-state functional connectivity and graph theory measures for each neurotransmitter network for MS patients. Linear regression was performed only for those networks that showed a difference at the between-group analysis. Age and gender were included in the analysis as covariates for the between-group comparison, while age, gender, cortical gray matter volume and T2 lesions volume were used as covariate for the linear regression analyses with the EDSS, SDMT, and HRSD as dependent variables. Only age and gender were included as covariates for the linear regression analyses with disease duration and disease severity as dependent variable, considering the close interaction between these two latter variables and both lesion volume and cortical atrophy. Given the exploratory nature of the study, a threshold of *P* < 0.01 uncorrected was applied for between-groups and linear regression data analyses. Notwithstanding the exploratory nature of the study, we also applied the Benjamini–Hochberg procedure to control for the false discovery rate when evaluating correlation matrices in the FC analysis, setting the false discovery rate to 10% [33].

## Results

### Clinical assessments

MS patients performed worse than HCs in the SDMT (*P* < 0.001) and scored higher in HRSD (*P* < 0.001). Clinical, demographic, and imaging characteristics of the HCs and MS patients are summarized in Table 1.

**Table 1:** Demographic, clinical and radiological features of Multiple Sclerosis patients and healthy controls

Characteristic	MS patients	Healthy Controls	*P* value
Subjects	29	24	–
Female sex, *N* (%)	17 (59)	15 (63)	0.8
Age, mean ± SD (years)	42.7 ± 8.2	38.2 ± 8.4	0.06
Disease duration, mean ± SD (years)	10.8 ± 1.5	–	–
EDSS, mean ± SD	3.2 ± 1.3	–	–
MSSS, mean ± SD	4.2 ± 2	–	–
Log.T2 lesional load (mm^3^), median (range)	3.47 (0.48–29.50)	–	–
Timed 25-foot walk (s), mean ± SD	20.7 ± 10.5	16.6 ± 6.01	0.20
Raw SDMT score, mean ± SD	40.6 ± 9.8	54.5 ± 9.66	< 0.001
HRSD, mean ± SD	6.39 ± 4.21	0.56 ± 0.96	< 0.001
*Disease modifying treatment*			
None, *N* (%)	7 (24)	–	
Interferon β1-a, *N* (%)	2 (7)	–	
Dimethyl fumarate, *N* (%)	7 (24)	–	
Teriflunomide, *N* (%)	1 (3.5)	–	
Fingolimod, *N* (%)	7 (24)	–	
Natalizumab, *N* (%)	1 (3.5)	–	
Alemtuzumab, *N* (%)	3 (10.5)	–	
Daclizumab, *N* (%)	1 (3.5)	–	

*N* number, *SD* standard deviation

### Functional connectivity in serotonergic–noradrenergic network

**Fig. 2:**
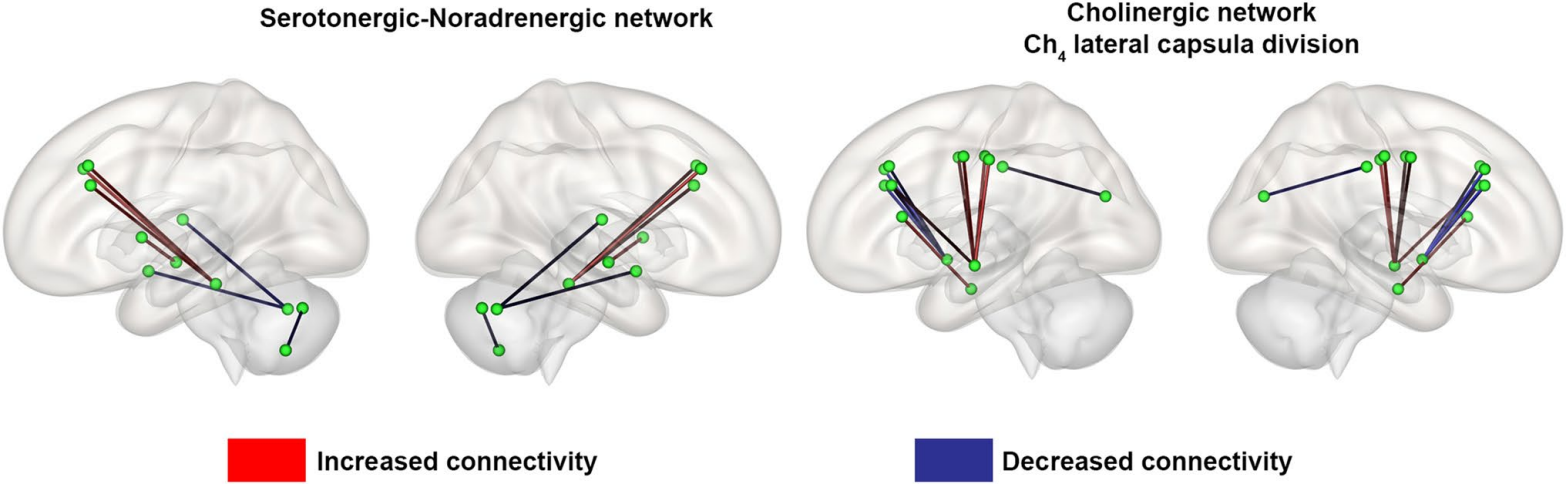
Resting-state functional connectivity changes in multiple sclerosis. Abnormalities of resting-state functional connectivity in multiple sclerosis (MS) patients compared to healthy controls (HCs) in the different neuromodulatory networks. Blue connections represent a decreased functional connectivity between the two regions of interest in MS patients compared to HCs. Red connections represent an increase functional connectivity between the two regions of interest in MS patients compared to HCs

MS patients showed decreased resting-state functional connectivity between different cerebellum regions (*P* = 0.006) and between the cerebellum and both the right amygdala and right thalamus (*P* < 0.01) compared with HCs. Patients also showed increased resting-state functional connectivity between the dorsal raphe and both the left and right superior frontal gyrus (*P* < 0.001), between the dorsal raphe and the left middle frontal gyrus, (*P* = 0.002), and between the left globus pallidus and the right hippocampus (*P* = 0.001) compared with HCs (Table 2 and Fig. 2).

**Table 2:** Brain areas showing significant differences of resting state functional MRI between healthy controls and multiple sclerosis patients within each neurotransmitter network

Neurotransmitter network	Source	Target	*T* value	*P *value	FDR-adjusted *P *value
Serotonergic–noradrenergic	Dorsal raphe	Superior frontal gyrus right	4.04	< 0.001	< 0.001*
		Superior frontal gyrus left	3.62	< 0.001	< 0.001*
		Middle frontal gyrus left	3.34	0.002	0.001
	Cerebellum-Crus I right	Amygdala right	− 2.76	0.008	0.002
		Thalamus right	− 2.71	0.009	0.002
	Cerebellum-Vermis Crus II	Cerebellum-Left VIIb	− 2.88	0.006	0.002
	Globus pallidus left	Hippocampus right	3.36	0.001	0.001
Cholinergic					
Ch_4_ lateral capsula division	Ch4 nucleus	Superior frontal gyrus left	− 3.51	0.001	< 0.001*
		Middle frontal gyrus right	− 3.47	0.001	0.001*
		Superior frontal gyrus right	− 3.01	0.004	0.002
		Middle frontal gyrus left	− 2.87	0.006	0.003
	Posterior cingulate gyrus left	Cuneus left	− 2.91	0.005	0.003
	Middle temporal gyrus left	Precentral gyrus left	3.47	0.001	0.001*
		Middle frontal gyrus left	3.3	0.002	0.002
		Postcentral gyrus right	3.22	0.002	0.002
		Precentral gyrus right	3	0.004	0.003
		Postcentral gyrus left	2.89	0.006	0.003
	Fusiform gyrus right	Inferior frontal gyrus left	2.73	0.009	0.004

Comparisons were performed using the contrast multiple sclerosis patients > healthy controls (two-tailed *t* test adjusted for age and gender, *P* < 0.01 uncorrected). *T* values are reported. Negative *T* values refer to a reduced connectivity between the source and target regions in multiple sclerosis patients compared to healthy controls

^*^Significant after FDR correction

**Fig. 3:**
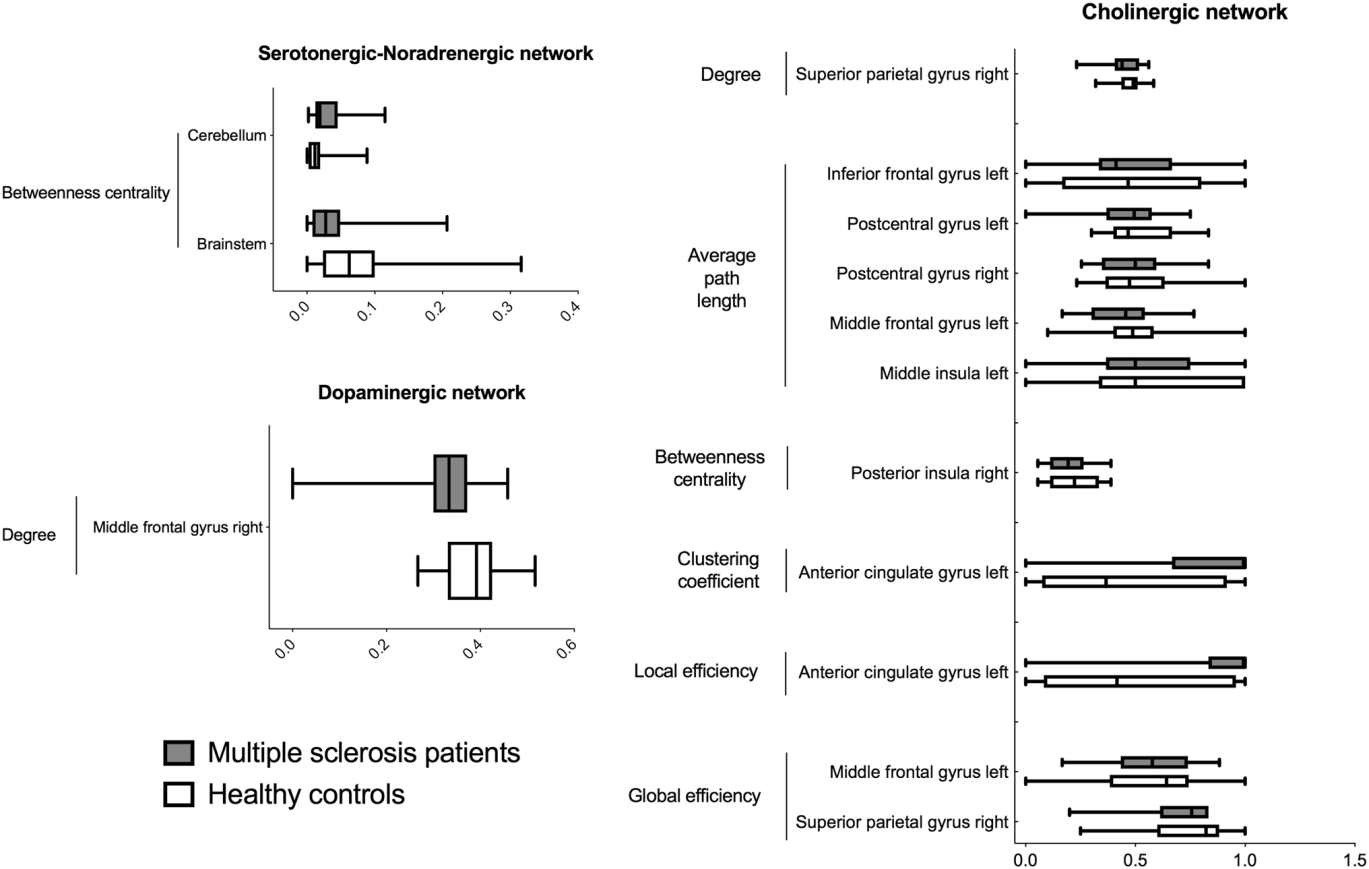
Graph theory measures between healthy controls and multiple sclerosis patients within each neuromodulatory networks. Brain areas showing significant differences for graph theory measures between healthy controls (HCs) (in black) and multiple sclerosis (MS) patients (in white) within the neuromodulatory networks. Comparisons were performed using the contrast MS patients > HCs (two-tailed *t *test adjusted for age and gender, *P* < 0.01 uncorrected). Data represent mean ± standard deviations

Graph theory analysis revealed that MS patients showed increased betweenness centrality in the cerebellum (*t*-value = 2.84; *P* = 0.007) and a decreased betweenness centrality in the brainstem (*t* value = − 3.03; *P* = 0.004) for the serotonergic–noradrenergic network (Table 3 and Fig. 3).

**Table 3:** Brain areas showing significant differences for graph theory measures between healthy controls and multiple sclerosis patients within each neurotransmitter network

Neurotransmitter network	Graph theory measure	Region of interest	*T* value	*P *value
Serotonergic–noradrenergic	Betweenness centrality	Brainstem	− 3.03	0.004
		Cerebellum-right VIIIa	2.84	0.007
Cholinergic				
Ch4 medial division	Local efficiency	Anterior cingulate gyrus left	0.36	0.006
	Clustering coefficient	Anterior cingulate gyrus left	0.34	0.007
Cholinergic				
Ch4 lateral perysilvian division	Betweenness Centrality	Posterior insula right	− 3.02	0.004
	Average path length	Middle insula left	− 2.94	0.005
Cholinergic				
Ch4 lateral capsula division	Global efficiency	Superior parietal gyrus right	− 2.85	0.006
		Middle frontal gyrus left	2.73	0.009
	Average path length	Middle frontal gyrus left	− 3.4	0.001
		Postcentral gyrus right	− 2.79	0.007
		Postcentral gyrus left	− 2.78	0.008
		Inferior frontal gyrus left	− 2.76	0.008
	Degree	Superior parietal gyrus right	− 2.85	0.006
Dopaminergic				
Motor division	Degree	Middle frontal gyrus right	− 2.86	0.006

Comparisons were performed using the contrast multiple sclerosis patients > healthy controls (two-tailed *t *test adjusted for age and gender, *P* < 0.01 uncorrected). *T *values are reported. Negative *t *values refer to a reduced value for the graph analysis measure in the selected region of interest in multiple sclerosis patients compared to healthy controls

### Functional connectivity in cholinergic network

#### *Ch*_*1-2-3*_* division*

Regarding Ch_1-2-3_ division of the cholinergic network, MS patients showed no differences for both functional connectivity and graph theory evaluation compared to HCs.

#### *Ch*_*4*_* medial division*

Functional connectivity evaluation for the Ch_4_ medial division of the cholinergic network showed no difference between HCs and MS patients. Graph theory analysis revealed that MS patients have a higher local efficiency (*t* value = 0.36; *P* = 0.006) and a higher clustering coefficient (*t*-value = 0.34; *P* = 0.007) compared with HCs for the Ch_4_ medial division of the cholinergic network (Table 3 and Fig. 3).

#### *Ch*_*4*_* lateral perisylvian division*

MS patients have a similar functional connectivity for the Ch_4_ lateral perisylvian division of the cholinergic network compared with HCs, whereas they showed a decreased betweenness centrality in the right insula (*t*-value = -3.02; *P* = 0.004) and a decreased average path length in the left insula (*t* value = -2.94; *P* = 0.005) compared with HCs when evaluating the network topography through graph theory (Table 3 and Fig. 3).

#### *Ch*_*4*_* lateral capsula division*

Resting-state fMRI evaluation revealed that MS patients, compared with HCs, have reduced functional connectivity between the Ch_4_ nucleus and both the middle and superior frontal gyrus (all *P* < 0.006), and between the left posterior cingulate cortex and the left cuneus (*t*-value = − 2.91; *P* = 0.005). MS patients also showed an increased connectivity between the left middle temporal gyrus and the right middle temporal gyrus (*t*-value = − 3.3; *P* = 0.002), between the left middle temporal gyrus and both the left and right pre- and post-central gyrus (all *P* < 0.006), and between the right fusiform gyrus and the left inferior frontal gyrus (*t*-value = 2.73; *P* = 0.009) (Table 2 and Fig. 2).

Graph theory assessment showed that MS patients have a reduced global efficiency in the right superior frontal gyrus (*t*-value = − 2.85; *P* = 0.006), a reduced average path length in the left middle frontal gyrus (*t*-value = − 3.4; *P* = 0.001), in the right and left postcentral gyrus (*t*-value = − 2.79; *P* = 0.008), and in the left inferior frontal gyrus (*t*-value = − 2.76; *P* = 0.008), and a reduced degree in the right superior frontal gyrus (*t*-value = − 2.85, *P* = 0.006) compared with HCs for the Ch_4_ lateral capsula division of the cholinergic network. MS patients also showed an increased global efficiency in the left middle frontal gyrus (*t*-value = 2.73; *P* = 0.009) (Table 3 and Fig. 3).

### Functional connectivity in dopaminergic network

#### Executive division

MS patients showed no differences for both functional connectivity and graph theory evaluation for the executive division of the dopaminergic network.

#### Limbic division

MS patients showed no differences for both functional connectivity and graph theory evaluation for the executive division of the dopaminergic network.

#### Motor division

MS patients showed no differences in resting-state functional connectivity when compared with HCs for the motor division of the dopaminergic network. Conversely, for the same network, graph theory analysis revealed that MS patients showed a decreased degree in the right middle frontal gyrus compared with HCs (*t*-value = − 2.86; *P* = 0.006) (Table 3 and Fig. 3).

### Clinical correlation analysis

Disease duration correlated with changes in the FC within the serotonergic–noradrenergic network (between cerebellum and right superior frontal gyrus, *t*-value = − 3.54, amygdala right and dorsal raphe, *t*-value = − 3.49, cerebellum and left inferior frontal gyrus, *t*-value = 3.54, right putamen and left globus pallidus, *t*-value = -2.92, cerebellum and right globus pallidus, *t*-value = − 3.29, ventral tegmental area and cerebellum, *t*-value = 3.54; all *P* < 0.01; Supplementary Table B-1), and the Ch_4_ lateral capsula division of the cholinergic network (between left inferior frontal gyrus and right amygdala, *t*-value = 3.67, right parahippocampal gyrus and left occipital lobe, *t*-value = − 2.87; all *P* < 0.01; Supplementary Table B-1). Similarly, disease duration also correlated with changes in the graph theory measures in the serotonergic–noradrenergic network, and the Ch_4_ lateral capsula and Ch_4_ lateral perisylvian divisions of the cholinergic network (all *P* < 0.01; Supplementary Table B-2). EDSS was associated with changes in FC within the serotonergic–noradrenergic network (between cerebellum and left middle frontal gyrus, *t*-value = − 3.75, right hippocampus and left putamen, *t*-value = − 3.04, right putamen and right hippocampus, *t*-value = - 3.52; all *P* < 0.01; Supplementary Table B-1), the Ch_4_ lateral capsula division of the cholinergic network (between left superior temporal gyrus and left cuneus, *t*-value = 3.18, and Ch_4_ nucleus and left angular gyrus, *t*-value = 3.25, right amygdala and right lingual gyrus, *t*-value = − 3.80; all *P* < 0.01; Supplementary Table B-1). EDSS was also associated with changes in the graph theory measures for the serotonergic–noradrenergic network, for the Ch_4_ lateral capsula division and Ch_4_ lateral perisylvian division of the cholinergic network, and for the motor division of the dopaminergic network (all *P* < 0.01; Supplementary Table B-2).

Disease severity, assessed with MSSS, correlated with changes in the FC in the serotonergic–noradrenergic network (between dorsal raphe and right amygdala, *t*-value = 3.60, left thalamus and left putamen, *t*-value = − 3.12, within the cerebellum, *t*-value = − 2.93; all *P* < 0.01; Supplementary Table B-1). Disease severity also correlated with changes in the graph theory measures for the serotonergic–noradrenergic network, for the Ch_4_ lateral perisylvian division of the cholinergic network, and for the motor division of the dopaminergic network (all *P* < 0.01) (Supplementary Table B-2).

Cognitive function, assessed with the SDMT, correlated with changes in the resting-state FC in the serotonergic–noradrenergic network (between right caudate nucleus and right inferior frontal gyrus, *t*-value = 4.25, cerebellum and left putamen, *t*-value = − 6.44, cerebellum and left hippocampus, *t*-value = − 4.04, right hippocampus and right caudate nucleus, *t*-value = 5.37, left hypothalamus and right putamen, *t*-value = 4.12, right inferior frontal gyrus and left superior frontal gyrus, *t*-value = 4.20, left putamen and left caudate nucleus, *t*-value = 4.04; right thalamus and cerebellum, *t*-value = − 4.19; locus coeruleus and right hippocampus, *t*-value = − 4.16; ventral tegmental area and left superior frontal gyrus, *t*-value = 6.71; all *P* < 0.01, Supplementary Table B-1), the Ch_4_ lateral capsular division of the cholinergic network (between right angular gyrus and left fusiform gyrus, *t*-value = − 4.52, right fusiform gyrus and right precentral gyrus, *t*-value = − 4.24, right posterior cingulate cortex and left occipital lobe, *t*-value = 6.60; all *P* < 0.01; Supplementary Table B-1), and the limbic division of the dopaminergic network (between left anterior cingulate cortex and left medial orbital gyrus, *t*-value = 5.77, left medial orbital gyrus and left anterior cingulate cortex, *t*-value = 3.90; all *P* < 0.01; Supplementary Table B-1). SDMT also correlated with changes in the graph theory measures for the serotonergic–noradrenergic network and for the Ch_4_ lateral perisylvian division of the cholinergic network (all *P* < 0.01; Supplementary Table B-2).

Depressive symptoms, assessed through the HRSD, were associated with changes in the resting-state FC for the serotonergic–noradrenergic network (between cerebellum and left nucleus accumbens, *t*-value = − 4.75, cerebellum and right hypothalamus, *t*-value = − 4.17, brainstem and left hypothalamus, *t*-value = − 5.53, cerebellum and right amygdala, *t*-value = 10.05, cerebellum and left hippocampus, *t*-value = − 4.14, cerebellum and right thalamus, *t*-value = 5.63; cerebellum and locus coeruleus, *t*-value = − 4.17; cerebellum and ventral tegmental area, *t*-value = 4.47; all *P* < 0.01; Supplementary Table B-1), and the Ch_4_ lateral capsula division of the cholinergic network (between Ch_4_ nucleus and right angular gyrus, *t*-value = 4.05, right amygdala and Postcentral gyrus left, *t*-value = 4.56, left amygdala and left supramarginal gyrus, *t*-value = 4.07; all *P* < 0.01; Supplementary Table B-1). HRSD correlated with changes in the graph theory measures for the serotonergic–noradrenergic network (all *P* < 0.01; Supplementary Table B-2).

## Discussion

Using a resting-state fMRI, we demonstrated FC changes and network topography derangements in serotonergic, noradrenergic, cholinergic, and dopaminergic systems. We highlighted that these changes has direct clinical implications resulting in both physical and cognitive disability in MS patients.

In MS, changes in network connectivity and topography within serotonergic, noradrenergic, cholinergic, and dopaminergic networks were not completely unexpected. First, the random occurrence of demyelinating lesions throughout the brain may cause white matter tract disconnection and brain reorganization, resulting in impaired FC. Considering the many long-range and diffuse projections to both cortical and subcortical regions from the relatively small cell populations that produce modulatory neurotransmitters (e.g., dorsal Raphe, Ch_4_ nucleus, and locus coeruleus), it is reasonable to expect that these neurochemical networks would be highly susceptible to white matter pathologies. Furthermore, given the wide range of physiological processes that these modulatory transmitter populations are implicated in it, quantification of functional changes in their associated networks could well provide a guide to the development and progression of clinical symptoms in MS. However, pathological changes lead to structural and functional reorganization of the brain to compensate for the loss of functions. Brain reorganization in MS is a dynamic process, likely characterized by increased FC as a compensatory mechanism during early stages and reduced ability to compensate for progressive brain damage at later stages, subsequently resulting in decreased FC [14]. Therefore, we cannot completely rule out the role of spatial reorganization of network anatomy in MS patients following brain damages. However, to partly overcome this issue, we used subject-specific regions of interest map, which allow us to take into account the subject anatomical variability and brain changes that might occur during the disease course, for example brain atrophy. Although ROI selection was performed to avoid brain regions overlapping, between the neuromodulatory networks due to the fact that in one single cortical area, we might find neuronal subpopulation belonging to different neuromodulatory networks, we cannot completely disentangle the role of each sub-population to the connectivity within the explored network. Notwithstanding this limitation, our exploratory study might drive new studies aimed at evaluating neuromodulatory network changes in MS taking advantage of newly developed PET tracers for neurotransmitters which are better equipped to dissociate neuromodulatory components in, for example, the dorsal raphe or cholinergic nuclei. Similarly, the reported alterations in FC might not necessarily imply a selective derangement in neuromodulatory networks, since they could reflect changes in non-neuromodulatory networks partly overlapping in terms of brain regions. Nonetheless these limitations, our results are consistent with the previous results. Hesse and colleagues reported reduced brain serotonin transporter levels in the cingulate cortex, the thalamus, and the insula, and a higher transporter availability in the orbitofrontal cortex in MS patients compared to HCs using PET molecular imaging with the radiotracer [^11^C]DASB [34]. In our study, we found reduced functional connectivity within the cerebellum, and between the cerebellum and both the amygdala and the thalamus, whereas there was an increased connectivity between the dorsal raphe and the frontal regions. In addition, we also outlined a decreased betweenness centrality for the brainstem in MS compared to HCs, suggesting that ascending projections from this structure are damaged and have a reduced activity in controlling the cortical regions of the network.

Differently for the serotonergic network, very little is known about noradrenalin deficits in MS. Noradrenalin concentration in the brain of MS patients was found to be either increased [35] or decreased [36]. The primary source of noradrenalin in the brain is the locus coeruleus [37]. Both inflammation and neurodegeneration have been described within the locus coeruleus of MS patients and the extent of neuronal damage shown to correlate with cognitive disability [38, 39]. Therefore, the reduced centrality of the brainstem in the noradrenalin network, reported in here, might be related to an overall reduced activity of the locus coeruleus with reduced function of the long-range projections heading to frontal regions and adaptive short-path intra-cerebellum adaptive connections, which increase the dysfunctional centrality of the cerebellum.

In our study, we also reported a functional disconnection between cortical and subcortical regions modulated by cholinergic neurons, with direct implications for cognition. Kimura and colleagues already reported tissue damage in the white matter bundles of the cholinergic network using a semi-quantitative measure and a correlation with cognitive impairment [40]. Finally, we also reported altered FC in the dopaminergic network in MS patients compared to HCs, showing a decreased connectivity between the ventral tegmental area and the orbital cortex and a decreased degree in the middle frontal gyrus. Up to now, no studies have specifically assessed dopaminergic network in MS. However, indirect report outlined the occurrence of white matter lesions over the bundles connecting the substantia nigra, the ventral tegmental areas, the striatum, the limbic areas, and the prefrontal cortex with changes in the local metabolic activity in the prefrontal cortex and in the striatum [41–43]. These damages may underpin FC changes and network reorganization, and are associated with cognitive impairment and fatigue [44–46].

Regarding clinical outcome measures, disease duration, disease severity, and physical disability were also associated at different extent with FC and network topography changes within serotonergic, cholinergic, dopaminergic, and noradrenergic network. We also reported a link between networks derangement and SDMT scores, suggesting a role for cognitive impairment. However, the SDMT is a single test assessing attention and information processing speed, thus exploring only a single cognitive domain. A more extensive cognitive battery, assessing different cognitive domains, such as the Brief Repeatable Battery of Neuropsychological Tests [47], could better elucidate the relationship between cognitive impairment and networks derangements. The association between network impairment and disease burden was evident especially for those networks with a wider distribution throughout the brain. This might be possibly due to a lower probability of demyelinating lesions over the connecting fiber bundles and subsequent disruption of neuron communication. Moreover, it is worth mentioning that both disease modifying and concomitant treatments, especially those aiming at providing relief from depressive symptoms, might impact on FC in neuromodulatory networks. The small sample in the present study prevents us from being able to draw conclusions on this topic. Further studies with larger samples, and targeted study design, are warranted to better evaluate the impact of disease modifying and concomitant therapies on FC changes in neuromodulatory networks. Therefore, the overall neurotransmitter network impairment might be used as a marker of brain damage and could be useful to stratify patients according to the severity of the disease.

In addition, we reported an association between depressive symptoms and both serotonergic and noradrenergic network topography reorganization. Our findings are in line with the reduction in the monoamine network activity, namely noradrenalin and serotonin, in major depressive disorder [48] and in MS [34]. In addition, we found that depression in MS was associated with an increased connectivity throughout the cholinergic network. This is in line with the previous findings describing an association between depressive symptoms and over-activity of the cholinergic system throughout the brain [49, 50]. Taken together, these findings suggest that depressive symptoms in MS are highly dependent upon neurotransmission integrity and an integrated therapeutic approach using drugs targeting different neurotransmitters might be useful. Moreover, depressive symptoms in MS include a broad spectrum of symptoms such as decreased concentration, fatigue, and sexual disinterest. Understanding better the neuronal basis of each of these symptoms might help clinicians towards more targeted symptomatic treatment choices to obtain a higher treatment efficacy.

## Conclusions

Through utilization of resting-state fMRI, and adopting an innovative method for reconstructing neuromodulatory networks maps in a subject-specific fashion, we revealed a diffuse FC impairment and topography derangements within the serotonergic, noradrenergic, cholinergic, and dopaminergic networks in MS with direct impact on clinical disability. This is the first study investigating neurotransmitter pathology in MS. Considering that resting-state fMRI might not disentangle FC changes specifically due to neuro-modulatory damages from those related to non-neuromodulatory connections, further studies assessing the impairment for each individual network using selective PET tracers are highly warranted to better understand the role of each network in disease pathophysiology and drive towards clinical applications of these findings. Neurotransmitter impairment might be a predictor of disability burden over time and might be used ideally to assess treatment efficacy. Moreover, confirmatory studies might also lead to the development of new symptomatic treatment based on neurotransmitter modulation.

## Electronic supplementary material

Below is the link to the electronic supplementary material.

Supplementary file1 includes an in-depth explanation on the methodology we used to set the network maps and additional tables containing ROI-based connectivity analysis (appendix A-1 and Table B-1 and B-2) and correlations with clinical outcome measures (DOCX 95 kb)

## Data Availability

Data will be made available via a request to the authors after a formal data sharing agreement.
