# Supplementary material for: Impaired connectivity within neuromodulatory networks in multiple sclerosis and clinical implications
Source: J Neurol. 2020 Mar 26;267(7):2042–53. doi: 10.1007/s00415-020-09806-3 (PMC7320961; doi:10.1007/s00415-020-09806-3)
Supplement: Supplementary file 1 — Supplementary file1 includes an in-depth explanation on the methodology we used to set the network maps and additional tables containing ROI-based connectivity analysis (appendix A-1 and Table B-1 and B-2) and correlations with clinical outcome measures (DOCX 95 kb) [file 415_2020_9806_MOESM1_ESM.docx]

**Article title:** Impaired connectivity within neuromodulatory networks in multiple sclerosis and clinical implications.

**Journal name:** Journal of Neurology

**Authors:** Antonio Carotenuto, Heather Wilson, Beniamino Giordano, Silvia P. Caminiti, Zachary Chappell, Steven C.R. Williams, Alexander Hammers, Eli Silber, Peter Brex, Marios Politis.

**Corresponding author:** Professor Marios Politis, Neurodegeneration Imaging Group, University of Exeter Medical School, London, United Kingdom; m.politis@exeter.ac.uk.

**Appendix A-1**

The serotonergic network was created following Strac and colleagues, and Jacobs and Azmitia guidelines (1, 2). Serotonergic network included the cerebellum from the probabilistic atlas for cerebellar lobules (3), the dorsal raphe, medial raphe and pontis oralis from the ascending arousal network atlas (4); hypothalamus from the CIC atlas version 2.0 (5); brainstem, putamen, caudate, thalamus, globus pallidus, nucleus accumbens, hippocampus, amygdala, inferior middle and superior frontal gyrus from the subject specific brain segmentation map generated using the multi-atlas propagation with enhanced registration approach (MAPER) (6). The noradrenergic network was created following Samuels and colleagues (7). It included the cerebellum from the probabilistic atlas for cerebellar lobules (3), the ventral tegmental area and peduncolopontine nucleus from the ascending arousal network atlas (4), hypothalamus from the CIC atlas version 2.0 (5), brainstem, hippocampus, amygdala, inferior, middle and superior frontal gyrus from the subject specific brain segmentation map generated using MAPER (6). Taking into account Sthe overlap for ROI falling within both serotonergic and noradrenergic networ we decided to merge the two networks creating a serotonergic-noradrenergic (7). The cholinergic network was created following Selden and colleagues, 1998 and divided in four different divisions: the Ch_1-2-3_ division, the Ch_4_ medial division, the Ch_4_ lateral perisylvian division and the Ch_4_ lateral capsula division (8). The Ch_1-2-3_ division included the Ch_1-2-3_ nuclei derived from the SPM anatomy toolbox (9) and all the brain regions receiving afferent fibres from them, namely the hypothalamus derived from the CIC atlas version 2.0 (5); the hippocampus derived from the subject specific brain segmentation map (MAPER (6)); and the olfactory cortex derived from the automated anatomical labelling atlas (10). The Ch_4_ medial division included the Ch_4_ nucleus derived from the SPM anatomy toolbox (9); and the anterior and posterior cingulate gyrus, the anterior, medial, lateral and posterior orbital gyrus and the rectus gyrus from the subject specific brain segmentation map (MAPER (6)). The Ch_4_ lateral perisylvian division included the Ch_4_ nucleus derived from the SPM anatomy toolbox (9); the fronto-pariental operculum, the Heschl's gyri and the olfactory cortex derived from the automated anatomical labelling atlas (10); and the superior temporal gyrus and the insula from the subject specific brain segmentation map (MAPER (6)). The Ch_4_ lateral capsula division included the Ch_4_ nucleus derived from the SPM anatomy toolbox (9); and the superior, middle and superior frontal gyrus, the pre- and post-central gyrus, the fusiform gyrus, the middle and inferior temporal gyrus, the superior temporal gyrus, the parahippocampal and ambient gyrus, the amygdala, the angular gyrus, the supramarginal gyrus, the superior parietal gyrus, the posterior cingulate gyrus, the lingual gyrus, the cuneus and the lateral occipital lobe from the subject specific brain segmentation map generated using MAPER (6).

The dopaminergic network was created following Tziortzi and colleagues, 2014 and divided in three different divisions: the dopaminergic executive division, the dopaminergic limbic division and the dopaminergic motor division (11). The dopaminergic executive division included the striatum executive area derived from the Oxford-GSK-Imanova connectivity striatal atlas (11), the inferior, middle and superior frontal gyrus from the subject specific brain segmentation map (MAPER; (6)). The dopaminergic limbic division included the striatum limbic area derived from the Oxford-GSK-Imanova connectivity striatal atlas (11), the anterior, medial and posterior orbital gyrus, the straight gyrus, the anterior cingulate cortex and the sub-callosal area from the subject specific brain segmentation map (MAPER; (6)). The dopaminergic motor division included the striatum motor area derived from the Oxford-GSK-Imanova connectivity striatal atlas (11), the substantia nigra, the inferior, middle and superior frontal gyrus and the pre-central gyrus from the subject specific brain segmentation map generated using MAPER (6).

**References**

1. Svob Strac D, Pivac N, and Muck-Seler D. The serotonergic system and cognitive function. *Transl Neurosci.* 2016;7(1):35-49.

2. Jacobs BL, and Azmitia EC. Structure and function of the brain serotonin system. *Physiol Rev.* 1992;72(1):165-229.

3. Diedrichsen J, Balsters JH, Flavell J, Cussans E, and Ramnani N. A probabilistic MR atlas of the human cerebellum. *Neuroimage.* 2009;46(1):39-46.

4. Edlow BL, Takahashi E, Wu O, Benner T, Dai G, Bu L, et al. Neuroanatomic connectivity of the human ascending arousal system critical to consciousness and its disorders. *J Neuropathol Exp Neurol.* 2012;71(6):531-46.

5. Tziortzi AC, Searle GE, Tzimopoulou S, Salinas C, Beaver JD, Jenkinson M, et al. Imaging dopamine receptors in humans with [11C]-(+)-PHNO: dissection of D3 signal and anatomy. *Neuroimage.* 2011;54(1):264-77.

6. Heckemann RA, Keihaninejad S, Aljabar P, Rueckert D, Hajnal JV, Hammers A, et al. Improving intersubject image registration using tissue-class information benefits robustness and accuracy of multi-atlas based anatomical segmentation. *Neuroimage.* 2010;51(1):221-7.

7. Samuels ER, and Szabadi E. Functional neuroanatomy of the noradrenergic locus coeruleus: its roles in the regulation of arousal and autonomic function part I: principles of functional organisation. *Curr Neuropharmacol.* 2008;6(3):235-53.

8. Selden NR, Gitelman DR, Salamon-Murayama N, Parrish TB, and Mesulam MM. Trajectories of cholinergic pathways within the cerebral hemispheres of the human brain. *Brain.* 1998;121 ( Pt 12):2249-57.

9. Eickhoff SB, Stephan KE, Mohlberg H, Grefkes C, Fink GR, Amunts K, et al. A new SPM toolbox for combining probabilistic cytoarchitectonic maps and functional imaging data. *Neuroimage.* 2005;25(4):1325-35.

10. Tzourio-Mazoyer N, Landeau B, Papathanassiou D, Crivello F, Etard O, Delcroix N, et al. Automated anatomical labeling of activations in SPM using a macroscopic anatomical parcellation of the MNI MRI single-subject brain. *Neuroimage.* 2002;15(1):273-89.

11. Tziortzi AC, Haber SN, Searle GE, Tsoumpas C, Long CJ, Shotbolt P, et al. Connectivity-based functional analysis of dopamine release in the striatum using diffusion-weighted MRI and positron emission tomography. *Cereb Cortex.* 2014;24(5):1165-77.

**Table B-1**. **Correlations of T scores from resting-state functional connectivity analyses with clinical and neuropsychological variables in multiple sclerosis patients.** Correlations were assessed after correction for age and gender, *P*<0.01 uncorrected. Positive T values represent positive correlations; negative T values represent negative correlations between resting-state functional connectivity in the two brain areas and clinical variables.

| **Clinical outcome** | **Neurotransmitter Network** | **Source** | **Target** | **T-Value** | **P-value** | **FDR adjusted P-value** |
| --- | --- | --- | --- | --- | --- | --- |
|  | **Serotonergic-Noradrenergic** | Cerebellum-left VIIIb | Superior frontal gyrus right | -3.54 | 0.002 | <0.001 |
| **Disease Duration** |  |  | Cerebellum-left V | 2.88 | 0.008 | 0.003 |
|  |  | Amygdala right | Dorsal raphe | -3.49 | 0.002 | <0.001 |
|  |  |  | Inferior frontal gyrus left | 3.48 | 0.002 | 0.001 |
|  |  | Cerebellum-right VIIb | Inferior frontal gyrus left | 3.54 | 0.002 | 0.001 |
|  |  | Putamen right | Globus pallidus left | -2.92 | 0.007 | 0.002 |
|  |  | Cerebellum-vermis X | Globus pallidus right | -3.29 | 0.003 | 0.002 |
|  |  | Ventral tegmental area | Cerebellum-left IX | -3.46 | 0.002 | 0.001 |
|  |  |  | Cerebellum-right IX | -3.12 | 0.005 | 0.002 |
|  | **Cholinergic** |  |  |  |  |  |
|  | **Ch4 lateral capsula division** | Inferior frontal gyrus left | Amygdala right | 3.67 | 0.001 | <0.001 |
|  |  | Parahippocampal gyrus right | Occipital lobe left | 2.87 | 0.008 | <0.001 |
| **Expanded Disability Status Scale** | **Serotonergic-Noradrenergic** | Cerebellum-right X | Cerebellum-left VIIIa | 3.33 | 0.003 | <0.001 |
|  |  |  | Cerebellum-vermis X | -3.28 | 0.003 | <0.001 |
|  |  | Cerebellum-left X | Middle frontal gyrus right | -3.52 | 0.002 | <0.001 |
|  |  | Cerebellum-left IX | Amygdala right | -3.05 | 0.005 | 0.001 |
|  |  | Cerebellum-vermis VIIIb | Amygdala left | -2.94 | 0.007 | 0.002 |
|  |  | Ventral tegmental area | Brainstem | 2.92 | 0.007 | 0.002 |
|  | **Cholinergic** |  |  |  |  |  |
|  | **Ch4 lateral capsula division** | Superior temporal gyrus left | Cuneus left | 3.15 | 0.004 | <0.001 |
|  |  | Angular gyrus right | Inferior frontal gyrus right | 3.32 | 0.003 | <0.001 |
|  | **Dopaminergic** |  |  |  |  |  |
|  | **Limbic division** | Posterior orbital gyrus right | Anterior cingulate gyrus left | -2.82 | 0.009 | 0.002 |
| **Multiple Sclerosis Severity Score** | **Serotonergic-Noradrenergic** | Dorsal raphe | Amygdala right | 3.6 | 0.001 | <0.001 |
|  |  |  | Cerebellum-right VI | 2.94 | 0.007 | <0.001 |
|  |  | Cerebellum-vermis VIIIb | Cerebellum-right crus I | -2.93 | 0.007 | 0.001 |
|  |  | Thalamus left | Putamen left | -3.12 | 0.004 | <0.001 |
| **Symbol Digit Modalities test** | **Serotonergic-Noradrenergic** | Thalamus left | Thalamus right | 6.02 | <0.001 | <0.001* |
|  |  |  | Caudate nucleus left | 3.78 | <0.001 | <0.001* |
|  |  |  | Caudate nucleus right | 3.75 | <0.001 | <0.001* |
|  |  | Cerebellum-vermis IX | Cerebellum-vermis VIIIb | 3.25 | 0.002 | 0.005* |
|  |  | Thalamus right | Caudate nucleus right | 4.66 | <0.001 | 0.001* |
|  |  |  | Cerebellum-left X | 3.55 | 0.001 | 0.002* |
|  |  |  | Caudate nucleus left | 3.47 | 0.001 | 0.003* |
|  |  | Globus pallidus right | Caudate nucleus right | 3.24 | 0.002 | 0.005* |
|  |  |  | Putamen left | 3.19 | 0.003 | 0.007* |
|  |  |  | Putamen right | 3.19 | 0.003 | 0.008* |
|  |  |  | Inferior frontal gyrus left | 2.84 | 0.007 | 0.01* |
|  |  |  | Hypothalamus left | 2.74 | 0.009 | 0.02* |
|  |  | Putamen left | Putamen right | 4.54 | <0.001 | 0.001* |
|  |  |  | Globus pallidus left | 3.2 | 0.003 | 0.008* |
|  |  |  | Caudate nucleus right | 2.79 | 0.009 | 0.002 |
|  |  | Globus pallidus left | Inferior frontal gyrus left | 3.35 | 0.002 | 0.005* |
|  |  | Cerebellum-left IX | Nucleus accumbens left | 3.22 | 0.003 | 0.008* |
|  |  |  | Nucleus accumbens right | 3.03 | 0.004 | 0.01* |
|  |  |  | Dorsal raphe | 2.81 | 0.008 | 0.01* |
|  |  | Hypothalamus right | Cerebellum-right crus II | 3.2 | 0.003 | 0.009* |
|  |  | Putamen right | Caudate nucleus right | 3.2 | 0.004 | 0.01* |
|  |  | Caudate nucleus right | Caudate nucleus left | 3.17 | 0.003 | 0.009* |
|  |  | Dorsal raphe | Superior frontal gyrus right | 3.58 | <0.001 | 0.002* |
|  |  |  | Brainstem | 3.32 | 0.002 | 0.006* |
|  |  |  | Middle frontal gyrus left | 3.27 | 0.002 | 0.006* |
|  |  |  | Cerebellum-right IX | 2.83 | 0.007 | 0.01* |
|  |  |  | Superior frontal gyrus left | 2.71 | 0.01 | 0.02* |
|  |  | Caudate nucleus left | Superior frontal gyrus left | 3.43 | 0.001 | 0.004* |
|  |  | Cerebellum-vermis VIIIa | Cerebellum-vermis VIIIb | 3.92 | <0.001 | 0.002* |
|  |  |  | Cerebellum-vermis crus II | 3.57 | 0.001 | 0.004* |
|  |  |  | Amygdala right | 3.03 | 0.004 | 0.01* |
|  |  | Amygdala right | Amygdala left | 3.34 | 0.002 | 0.007* |
|  |  | Middle frontal gyrus left | Superior frontal gyrus left | 3.9 | 0.004 | 0.01* |
|  |  | Middle frontal gyrus right | Superior frontal gyrus right | 3.27 | 0.002 | 0.007* |
|  |  | Amygdala left | Median raphe | -3.08 | 0.004 | 0.01* |
|  |  | Pontis oralis | Hypothalamus left | 2.84 | 0.007 | 0.01* |
|  |  |  | Median raphe | 2.84 | 0.007 | 0.01* |
|  |  | Cerebellum-right crus I | Cerebellum-right crus II | 2.81 | 0.008 | 0.02* |
|  |  | Cerebellum-left I-IV | Cerebellum-right V | 2.86 | 0.007 | 0.01* |
|  |  | Cerebellum-vermis VI | Cerebellum-left VIIb | -2.77 | 0.009 | 0.02* |
|  |  | Cerebellum-right V | Cerebellum-left V | 3.05 | 0.004 | 0.01* |
|  |  | Nucleus accumbens left | Cerebellum-left VI | 3.17 | 0.003 | 0.01* |
|  |  | Ventral tegmental area | Middle frontal gyrus left | -2.86 | 0.0068 | 0.02* |
|  |  |  | Middle frontal gyrus right | -3.03 | 0.0043 | 0.01* |
|  |  | Peduncolo-pontine nucleus | Cerebellum-vermis VIIb | 2.88 | 0.0064 | 0.01* |
|  | **Cholinergic** |  |  |  |  |  |
|  | **Ch4 lateral capsula division** | Amygdala right | Amygdala left | 3.26 | 0.0023 | 0.005* |
|  |  | Angular gyrus right | Superior parietal gyrus right | 3.15 | 0.0031 | 0.006* |
|  |  | Lingual gyrus left | Lingual gyrus right | 4.82 | <0.001 | <0.001* |
|  |  |  | Cuneus right | 4.09 | <0.001 | <0.001* |
|  |  | Lingual gyrus right | Cuneus right | 3.52 | 0.0011 | 0.002* |
|  |  |  | Occipital lobe left | 3.13 | 0.0033 | 0.006* |
|  |  | Middle frontal gyrus left | Superior frontal gyrus left | 3.9 | <0.001 | 0.001* |
|  |  | Middle frontal gyrus right | Ch4 nucleus | -2.89 | 0.0063 | 0.001* |
|  |  |  | Angular gyrus right | 2.73 | 0.0094 | 0.007 |
|  |  | Middle temporal gyrus left | Middle temporal gyrus right | 3.59 | 0.0009 | 0.002* |
|  |  |  | Amygdala right | 2.87 | 0.0066 | 0.007* |
|  |  | Parahippocampal gyrus left | Fusiform gyrus right | 3.88 | 0.0004 | <0.001* |
|  |  |  | Fusiform gyrus left | 3.29 | 0.0021 | 0.004* |
|  |  |  | Parahippocampal gyrus right | 3.52 | 0.0011 | 0.002* |
|  |  | Parahippocampal gyrus right | Fusiform gyrus right | 3.3 | 0.002 | 0.004* |
|  |  | Postcentral gyrus right | Postcentral gyrus left | 3.19 | 0.0028 | 0.005* |
|  |  | Posterior cingulate gyrus right | Posterior cingulate gyrus left | 3.53 | 0.0011 | 0.003* |
|  |  | Precentral gyrus left | Precentral gyrus right | 3.62 | 0.0008 | 0.001* |
|  |  | Superior frontal gyrus right | Middle frontal gyrus left | 3.27 | 0.0023 | 0.005* |
|  | **Dopaminergic** |  |  |  |  |  |
|  | **Limbic division** | Anterior cingulate gyrus left | Anterior cingulate gyrus right | 4.38 | <0.001 | 0.002* |
|  |  |  | Posterior Orbital gyrus left | 3.36 | 0.002 | <0.001 |
|  |  | Anterior cingulate gyrus right | Posterior Orbital gyrus left | 2.92 | 0.006 | 0.002 |
|  |  |  | Posterior Orbital gyrus right | 2.81 | 0.008 | 0.002 |
|  |  | Rectus gyrus right | Rectus gyrus left | 3.79 | <0.001 | 0.001* |
|  |  |  | Medial orbital gyrus right | 3.02 | 0.004 | 0.002 |
|  |  | Subcallosal area right | Subcallosal area left | 3.19 | 0.003 | 0.001 |
| **Hamilton rating scale for depression** | **Serotonergic-Noradrenergic** | Middle frontal gyrus left | Cerebellum-right VIIb | -3.61 | 0.0008 | <0.001 |
|  |  |  | Cerebellum-vermis IX | 3.08 | 0.0037 | <0.001 |
|  |  | Cerebellum-right X | Cerebellum-left crus I | -3.52 | 0.0011 | <0.001 |
|  |  |  | Dorsal Raphe | 2.92 | 0.0058 | 0.001 |
|  |  | Thalamus right | Cerebellum-right VIIIb | 2.97 | 0.005 | 0.001 |
|  |  | Cerebellum-vermis VIIb | Cerebellum-right VIIIa | -2.91 | 0.0058 | 0.002 |
|  |  | Superior frontal gyrus left | Cerebellum-right VIIb | -2.88 | 0.0064 | 0.002 |
|  |  | Nucleus accumbens right | Hypothalamus left | 2.85 | 0.0069 | 0.002 |
|  | **Cholinergic** |  |  |  |  |  |
|  | **Ch4 lateral capsula division** | Superior parietal gyrus left | Superior temporal gyrus left | 3.68 | <0.001 | <0.001 |
|  |  |  | Amygdala left | 2.89 | 0.006 | <0.001 |
|  |  | Superior temporal gyrus left | Lingual gyrus right | 2.8 | 0.008 | 0.002 |
|  |  | Ch4 nucleus | Inferior frontal gyrus left | 2.96 | 0.005 | <0.001 |
|  |  |  | Middle frontal gyrus left | 2.85 | 0.007 | 0.001 |
|  |  | Parahippocampal gyrus left | Posterior cingulate gyrus right | 2.76 | 0.009 | 0.002 |
|  | **Dopaminergic** |  |  |  |  |  |
|  | **Limbic division** | Medial orbital gyrus left | Subcallosal area right | 3.01 | 0.004 | 0.002 |

*Significant correlation after FDR correction

**Table B-2**. Correlations of T scores from graph theory analyses with clinical and neuropsychological variables in multiple sclerosis patients. Correlations were assessed after correction for age and gender. *P*<0.01 uncorrected. Positive T values represent positive correlations; negative T values represent negative correlations between resting-state functional connectivity in the two brain areas and clinical variables.

| **Clinical outcome** | **Neurotransmitter network** | **Graph theory measure** | **Region of interest** | **T Value** | ***P* Value** |
| --- | --- | --- | --- | --- | --- |
| **Disease Duration** | **Serotonergic-Noradrenergic** | **Global efficiency** | Hypothalamus right | -3.67 | 0.001 |
|  |  |  | Dorsal raphe | -2.89 | 0.008 |
|  |  | **Local efficiency** | Putamen right | -4.05 | <0.001 |
|  |  | **Cluster coefficient** | Putamen right | -3.44 | 0.002 |
|  |  | **Degree** | Hypothalamus right | -3.42 | 0.002 |
|  |  |  | Dorsal raphe | -3.36 | 0.002 |
|  |  |  | Inferior frontal gyrus left | 2.97 | 0.006 |
|  |  | **Betweenness Centrality** | Inferior frontal gyrus left | 3.44 | 0.002 |
|  | **Cholinergic** |  |  |  |  |
|  | Ch_4_lateral perysilvian division | **Global efficiency** | Insula anterior pole right | -2.94 | 0.007 |
|  |  | **Average path length** | Insula anterior right | 3.08 | 0.006 |
|  | Ch_4_ lateral capsula division | **Betweenness Centrality** | Precentral gyrus left | 3.00 | 0.006 |
|  | **Serotonergic-Noradrenergic** | **Local efficiency** | Cerebellum | -3.16 | 0.006 |
|  |  | **Betweenness Centrality** | Cerebellum | 3.02 | 0.006 |
|  |  | **Clustering coefficient** | Cerebellum | -3.32 | 0.005 |
|  |  | **Degree** | Cerebellum | -3.13 | 0.005 |
| **Expanded disability status scale** | **Cholinergic** |  |  |  |  |
|  | Ch_4_lateral perysilvian division | **Local efficiency** | Anterior insula left | -3.54 | 0.003 |
|  |  |  | Anterior insula right | -3.09 | 0.009 |
|  |  | **Clustering coefficient** | Anterior insula left | -4.39 | 0.001 |
|  |  |  | Anterior insula right | -3.40 | 0.004 |
|  | Ch_4_ lateral capsula division | **Betweenness Centrality** | Postcentral gyrus right | 3.14 | 0.005 |
|  | **Dopaminergic** |  |  |  |  |
|  | Motor division | **Betweenness Centrality** | Middle frontal gyrus right | 0.03 | <0.001 |
| **Multiple sclerosis severity score** | **Serotonergic-Noradrenergic** | **Global efficiency** | Nucleus accumbens right | 2.80 | 0.010 |
|  |  |  | Dorsal raphe | 2.96 | 0.007 |
|  |  | **Betweenness centrality** | Cerebellum | 2.99 | 0.006 |
|  |  |  | Inferior frontal gyrus left | -2.82 | 0.009 |
|  |  | **Degree** | Nucleus accumbens right | 3.37 | 0.002 |
|  |  |  | Dorsal raphe | 3.16 | 0.004 |
|  | **Cholinergic** |  |  |  |  |
|  | Ch_4_ lateral perysilvian division | **Local efficiency** | Anterior insula left | -3.94 | <0.001 |
|  |  | **Cluster coefficient** | Anterior insula left | -3.62 | 0.002 |
|  | **Dopaminergic** |  |  |  |  |
|  | Motor division | **Global efficiency** | Middle frontal gyrus left | 3.17 | 0.004 |
|  |  | **Local efficiency** | Middle frontal gyrus left | -3.49 | 0.002 |
|  |  | **Betweenness centrality** | Middle frontal gyrus left | 3.05 | 0.005 |
|  |  | **Cluster coefficient** | Middle frontal gyrus left | -3.58 | 0.001 |
| **Symbol digit modalities test** | **Serotonergic-Noradrenergic** | **Global efficiency** | Cerebellum | -3.89 | 0.008 |
|  |  | **Betweenness Centrality** | Cerebellum | 0.09 | 0.005 |
|  |  | **Average path length** | Hypothalamus left | 13.32 | 0.006 |
|  |  |  | Cerebellum | 4.17 | 0.006 |
|  |  |  | Inferior frontal gyrus right | 3.72 | 0.010 |
|  |  | **Local efficiency** | Superior frontal gyrus right | 5.55 | 0.001 |
|  |  | **Clustering Coefficient** | Globus pallidus right | 4.03 | 0.010 |
|  | **Cholinergic** |  |  |  |  |
|  | Ch_4_ lateral perysilvian division | **Clustering coefficient** | Superior temporal gyrus | -7.11 | 0.006 |
| **Hamilton rating scale for depression** | **Serotonergic-Noradrenergic** | **Local efficiency** | Thalamus left | -6.61 | 0.003 |
|  |  | **Average path length** | Hypothalamus left | -7.93 | 0.004 |
|  |  | **Betweenness centrality** | Thalamus left | 4.31 | 0.008 |
|  |  | **Degree** | Cerebellum | -4.38 | 0.007 |
